# Luminescent Nanocrystal Probes for Monitoring Temperature and Thermal Energy Dissipation of Electrical Microcircuit

**DOI:** 10.3390/nano14241985

**Published:** 2024-12-11

**Authors:** Dawid Jankowski, Kamil Wiwatowski, Michał Żebrowski, Aleksandra Pilch-Wróbel, Artur Bednarkiewicz, Sebastian Maćkowski, Dawid Piątkowski

**Affiliations:** 1 Institute of Physics, Faculty of Physics, Astronomy and Informatics, Nicolaus Copernicus University in Toruń, ul. Grudziądzka, 5, 87-100 Toruń, Poland; dawid.jankowski@umk.pl (D.J.); m.zebrowski@umk.pl (M.Ż.); mackowski@fizyka.umk.pl (S.M.); 2 Institute of Low Temperature and Structure Research, Polish Academy of Sciences, ul. Okólna, 2, 50-422 Wroclaw, Poland; a.pilch@intibs.pl (A.P.-W.); a.bednarkiewicz@intibs.pl (A.B.)

**Keywords:** luminescent thermometry, thermal management, luminescent microscopy, up-conversion nanocrystals, erbium ions

## Abstract

In this work, we present an experimental approach for monitoring the temperature of submicrometric, real-time operating electrical circuits using luminescence thermometry. For this purpose, we utilized lanthanide-doped up-converting nanocrystals as nanoscale temperature probes, which, combined with a highly sensitive confocal photoluminescence microscope, enabled temperature monitoring with spatial resolution limited only by the diffraction of light. To validate our concept, we constructed a simple model of an electrical microcircuit based on a single silver nanowire with a diameter of approximately 100 nm and a length of about 50 µm, whose temperature increase was induced by electric current flow. By driving electric current only along one half of the nanowire, we created a dual-function microstructure, where one section is a resistive heater, while the other operates as a radiator. Such a combination realistically reflects the electronic circuit and its thermal behavior. We demonstrated that nanocrystals distributed around this circuit allow for remote temperature readout and enable precise monitoring of the thermal energy propagation and heat dissipation processes, which are crucial for designing and developing highly integrated electronic on-chip devices.

## 1. Introduction

Precise knowledge of the temperature of physical systems plays a crucial role in understanding and optimizing the processes ongoing in multiple fields of science and technology. As we approach the technological limits of electronic system miniaturization, there is an increasing need for precise temperature monitoring at the submicroscale and even nanoscale. Since the flow of electric current is inherently accompanied by the generation of heat energy via the Joule process, identifying and eliminating micro-damages, corrosion, and other unwanted defects arising during the manufacturing and assembly of electronic devices—which intensify the heating—are crucial for improving their functionality and durability [1]. The high densities of heat generated by densely packed electronic and optoelectronic systems further necessitate efficient heat dissipation mechanisms (thermal management). This is particularly important for systems operating under extreme conditions, including heavy industry, space applications, and military contexts [2].

One of the most exciting and rapidly developing techniques for temperature monitoring is luminescence thermometry. Its principle relies on the remote (non-contact) measurement of specific spectroscopic features of emitters as a function of temperature. Depending on the chosen method, this may involve analyzing the intensity ratios of the emission lines, their spectral shifts, or the luminescence decay times [3]. Various materials, including quantum dots, rare-earth-doped phosphors, and organic dyes, can serve as luminescent thermometers, each tailored to specific applications based on factors such as sensitivity, operational range, and stability. Luminescence thermometry can be applied across an extremely broad temperature range and operates effectively in challenging environments, such as high-pressure conditions or chemically reactive atmospheres, where conventional thermometry may fail [4]. A unique aspect of this technique concerns its almost unlimited scalability. Depending on the luminophore, luminescence thermometry can be used in both macroscopic (bulk crystals) and microscopic (nanocrystals) systems, with many approaches being discussed and optimized for industrial [5,6,7] and bio-medical applications [8,9,10]. Additionally, this method is non-invasive owing to the minimal size of the probe in comparison to the object of study. For instance, the thermal capacity of individual nanosensors is practically negligible, which is critical for applications in nanotechnology [11]. When combined with optical microscopy, luminescence thermometry enables real-time and in situ temperature monitoring with submicrometer resolution.

An exciting class of luminescent materials suitable for temperature monitoring at the nanoscale are fluoride nanocrystals (NCs) doped with rare-earth (RE) ions [12]. Compared to dye molecules [13] or quantum dots [14], RE-doped NCs are characterized by much narrower absorption and emission lines, which allows for simple extraction of temperature dependent spectral features. Moreover, owing to the forbidden nature of the f↔f optical transitions occurring in RE dopants, temperature-dependent luminescence decay times remain within the range of tens to hundreds of microseconds [15]. The latter requires relatively simple and inexpensive excitation and measuring equipment compared to nanosecond lifetimes of quantum dots or organic dyes. Furthermore, these RE-doped nanocrystals can often be excited through the sequential absorption of several low-energy infrared photons (up-conversion). This is advantageous for biological applications as this excitation falls within the spectral range of biological optical windows, ensuring high transparency in organic tissues [16,17]. Multiphoton excitation with infrared photons can lead to anti-Stokes emission in the visible range [18], which is spectrally separated from any laser noise or sample autofluorescence, thus yielding very high signal-to-noise ratios (SNRs). Years of observations have proven that fluoride nanocrystals doped with RE ions are also chemically stable, inherently non-toxic, and resistant to optical degradation [19].

**Figure 1:**
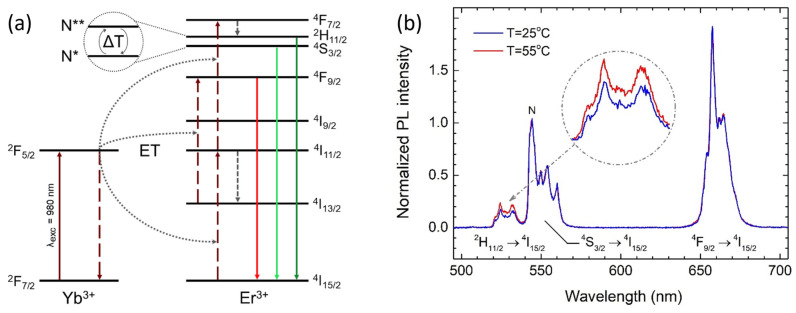
(**a**) Illustration of the energy diagram and up-conversion mechanism in nanocrystals activated by Er^3+^ and Yb^3+^ ions. Note the small energy gap between the ^4^S_3/2_ and ^2^H_11/2_ levels, whose relative populations can be thermally modified. Excited populations of these levels are denoted as N* and N**, respectively. (**b**) Example emission spectrum of the examined nanocrystal dense layer at temperatures of 25 °C and 55 °C. The spectra were normalized at 545 nm (N).

Among the most commonly used temperature-sensitive luminescent nanosensors are NaYF_4_ nanocrystals activated by Er^3+^ and Yb^3+^ ions. Due to the significant presence of ytterbium ions (sensitizers), these NCs exhibit highly efficient absorption of near-infrared excitation radiation, peaking at around 980 nm (^2^F_7/2_ → ^2^F_5/2_), which activates the up-conversion emission of erbium ions (Figure 1a). The energy absorbed by Yb^3+^ is non-radiatively transferred to Er^3+^, leading to the sequential absorption of two energy quanta—^4^I_15/2_ → ^4^I_11/2_ + ^4^I_13/2_ → ^4^F_9/2_ or ^4^I_15/2_ → ^4^I_11/2_ + ^4^I_11/2_ → ^4^F_7/2_—which activates visible emission. Typically, a single emission band peaking at 650 nm (^4^F_9/2_ → ^4^I_15/2_) is observed alongside two closely located bands around 550 nm (^4^S_3/2_ → ^4^I_15/2_) and 525 nm (^2^H_11/2_ → ^4^I_15/2_). The latter are commonly referred to as the S-band and H-band, respectively, and are widely explored in luminescent thermometry [20].

Due to the small energy gap (ΔE) between the levels ^4^S_3/2_ and ^2^H_11/2_ (several hundred cm^−1^ [21]) and their relatively long luminescence lifetimes (~200 µs [22]), their relative populations can be thermally modified and are well described by the Boltzmann distribution [23]. Consequently, based on the analysis of the relative intensities of the S and H emission bands, it is possible to determine the temperature, T, of the sensor with a sensitivity of about 1% K^−1^ using the equation

(1) $$T={\left(\ln\left(\frac{I_{S}}{I_{H}}\right)+C\right)}^{-1}$$

where k_B_ is the Boltzmann constant and *C* is a calibration constant characteristic of a given type of nanocrystal. The calibration process for the nanocrystals used in this work and the resulting parameterization of Equation (1) have been described previously [24].

The influence of temperature on the luminescent nanocrystals investigated in this work, when deposited as a high-density layer on a glass substrate, is shown in Figure 1b. A comparison of the normalized photoluminescence spectra of Er^3+^ measured at temperatures of 25 (blue) and 55 °C (red) shows that the relative intensity of the transitions ^4^F_9/2_ → ^4^I_15/2_ and ^4^S_3/2_ → ^4^I_15/2_ is independent of temperature. In contrast, the intensity of the transition ^2^H_11/2_ → ^4^I_15/2_ increases with temperature, which is in agreement with the mechanism described above.

In this work, we used Er^3+^-activated NCs to monitor the thermal properties of a model electrical microcircuit. Referring to previously reported results where micro- or nanocrystals were used to measure the temperature of resistively heated, relatively large microcircuits [25,26], our system was built around a single silver nanowire (NW). The diameter of the nanowire, which is approximately 100 nm, closely resembles the electrical connections used in modern integrated circuits. We present and discuss the applicability of the NCs for assessing the temperature of such a submicroscopic circuit, as well as for monitoring the heat removal and thermal energy dissipation processes with the nanoscale conductor (nanowire), which can be considered a radiator. Using confocal microscopy, we achieved resolution and sensitivity levels sufficient for temperature monitoring with individual nanocrystals (NCs) at the nanoscale.

## 2. Methodology

### 2.1. Luminescence Probes

Rare-earth-doped NaYF_4_ nanocrystals were prepared using a wet chemistry method consisting of two sequential processes. First, the cores of the NCs, containing 2 mol% Er^3+^ and 20 mol% Yb^3+^, were synthesized following the previously described protocol [27,28]. The resulting nanoparticles already exhibited the desired optical activity. However, to limit the interaction of the core with the environment—particularly with surfactants that can cause luminescence quenching—they were coated with a passive shell made from the same undoped matrix [29]. Ultimately, core–shell NCs dispersed in chloroform that are not susceptible to external chemical factors were obtained. The morphology of the synthesized NCs was examined using Transmission Electron Microscopy (TEM) and X-Ray Diffraction (XRD) methods. Statistical analysis revealed an average core diameter of 22 nm and a shell thickness of approximately 9 nm (see Supplementary Materials). Their crystal structure was determined using the International Centre for Diffraction Data (ICDD) database and identified as a pure hexagonal phase of a NaYF_4_ crystal (ICDD no. 04-011-3581) (see Supplementary Materials).

### 2.2. Model Electrical Circuit

**Figure 2:**
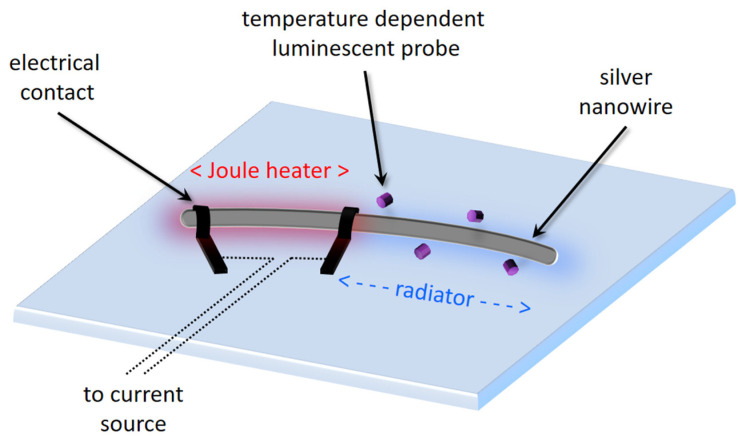
Conceptual diagram of the model electrical microcircuit illustrating the configuration of the heating element and the radiator. Objects are not to scale.

The model electrical microcircuit was prepared based on crystalline silver nanowires. These NWs were synthesized using a wet chemistry technique known as the polyol process [30] and subsequently characterized using TEM and Scanning Electron Microscopy (SEM). The nanowires exhibit a monocrystalline structure, with diameters typically ranging from 100 to 150 nm and maximum lengths of up to 50 µm (see Supplementary Materials). The model microcircuit consists of a single silver NW, to which two contacts were attached and linked to an external current source, as shown in Figure 2. The electric current flowing through the NW generates thermal energy via the Joule heating process, simulating the operation of a real electronic system. Notably, only a section of the nanowire functions as a microscopic heater (marked as “Joule heater” in Figure 2), as defined by the position of the second (middle) contact, which closes the electrical circuit roughly in the middle of the NW. The remaining part of the nanowire is not powered but participates in the conduction and dissipation of thermal energy from the heater (marked as “radiator” in Figure 2), where both parts mimic more complex electronic circuits.

### 2.3. Experimental Setup

**Figure 3:**
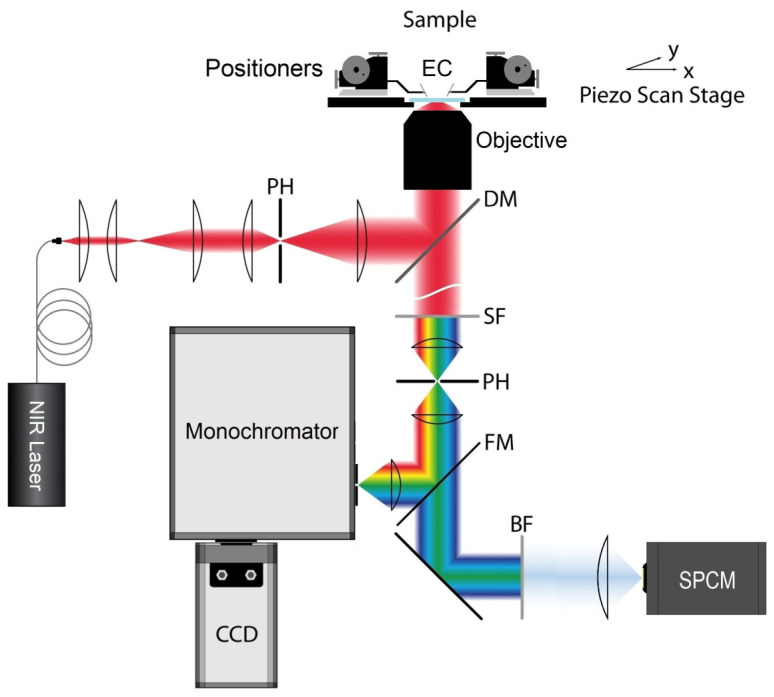
A simplified block diagram of the experimental setup. The components are labeled as follows: EC—electrical contact; DM—dichroic mirror; PH—diaphragm (pinhole); SF—short-pass filter; FM—flip mirror; BF—band-pass filter; and SPCM—single-photon counting module.

All experiments were performed using an inverted optical microscope (Eclipse Ti-S, Nikon, Tokyo, Japan) that allows for both wide-field sample preview and advanced confocal photoluminescence imaging (Figure 3). The microscope is equipped with a high numerical aperture objective (Plan Apo 60x, NA = 1.40, Nikon, Tokyo, Japan), which provides high spatial resolution for imaging and detection sensitivity, enabling the spectral and time-resolved characterization of single nanocrystals. The excitation source was a pig-tailed semiconductor laser diode (DBR976PN, Thorlabs, Newton, NJ, USA) integrated with a single-mode optical fiber, emitting radiation at 976 nm with a maximum power of approximately 30 mW. The collimated laser beam was reflected by a dichroic mirror (675dcspxr, Chroma, Foothill Ranch, CA, USA) and directed toward the objective, forming a diffraction-limited excitation spot with a diameter of approximately 430 nm. The same objective collected the light emitted (for photoluminescence imaging) or reflected (for backscattering imaging) from the sample, which then passed through a confocal aperture (d = 25 µm). Finally, the luminescence was redirected to either a single-photon counting module (SPCM, COUNT-100, Laser Components, Olching, Germany) or to a monochromator (Shamrock 500i, Oxford Instruments, Oxford, UK) coupled with an electron-multiplying CCD camera (Newton 940 CCD, Oxford Instruments, UK). The sample was mounted on a piezoelectric stage (P-517, Physik Instrumente, Karlsruhe, Germany) synchronized with the SPCM and a pulse counter (PCI-6236, National Instruments, Austin, TX, USA) for fast raster imaging. Additionally, two micropositioners equipped with tungsten electric probes (DPP220, Cascade Microtech, Beaverton, OR, USA) were mounted on the sample holder and connected to an external power supply (DP831A, Rigol, Suzhou, China) to control the current driven through the electrical microcircuit.

## 3. Experiment and Discussion

### 3.1. Preparation of the Electrical Microcircuit

**Figure 4:**
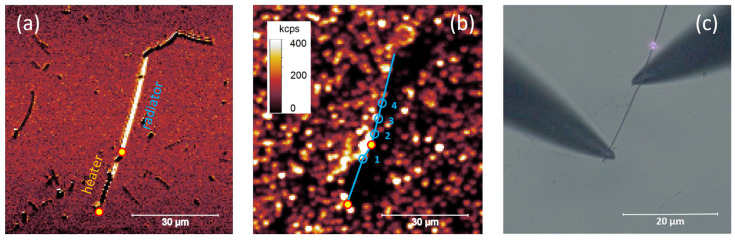
(**a**) A selected silver nanowire imaged in backscattered laser light, with the heating element and radiator indicated, where 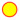 represents electrical contacts. (**b**) The same sample area captured in photoluminescence mode, showing the distribution of luminescent probes (nanocrystals) and the temperature readout positions 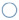. (**c**) A microscope image of the nanowire connected to an external power supply, where the laser spot (pink) indicates the position of the sample over the objective.

The initial phase of the experiment focused on preparing a model electrical microcircuit designed to simulate an operating electronic microdevice. First, a small volume (20 µL) of low-concentration colloidal NWs were spin-coated at 5000 rpm for 60 s onto a glass microscope cover slip. The resulting surface density of the nanowires was approximately one nanowire per 100 µm^2^. The main component of the microcircuit was a carefully selected single NW, about 50 µm in length, imaged using backscattered laser light, as shown in Figure 4a. The high quality of this nanowire was verified experimentally by monitoring the propagation length of laser-launched surface plasmon polaritons [31]. In the subsequent step, the sample was coated with a layer of luminescent nanoprobes—temperature indicators. To achieve this, 20 µL of nanocrystals suspended in chloroform were spin-coated at 2500 rpm for 30 s onto a NW-containing substrate. This process produced a relatively high surface density of nanocrystals—approximately 20 NCs per 100 µm^2^, as observed in photoluminescence imaging (Figure 4b). Such a relatively high surface density of luminescent probes was essential for mapping temperature around the circuit. At the end of this process, electrical probes were connected to the NW. The probes were positioned near the NW and gradually lowered while monitoring the contact moment using real-time microscope preview and by measuring the circuit resistance. For nanowires with a length of 20 µm, the circuit resistance was approximately 5–10 Ω, which was sufficient to achieve resistive heating. The positions of the probes—coupled to the left end and the middle of the NW—defined the heating section (approximately 20 µm long) and the radiator (around 30 µm long). A selected nanowire connected to the electrical probes is shown in Figure 4c.

### 3.2. Monitoring Temperature and Thermal Energy Dissipation

The electrical circuit shown in Figure 4 was subjected to a series of experiments that involved heating the NW through the passage of electric current. Based on numerous trials, we determined that a maximal, safe current intensity was approximately 15 mA for a 40–50 µm long nanowire. Higher current intensities typically resulted in irreversible damage to the NW and were therefore avoided. We did not observe any effect of laser irradiation on the conductive properties of the nanowire. Nanocrystals located in the heater part (Position 1) and the radiator part (Positions 2–4), observed at distances smaller than the laser spot diameter from the NW (Figure 4b), were used to assess the temperature of the microcircuit. The average distance between consecutive temperature readout positions was approximately 5 µm, measured along the NW.

**Figure 5:**
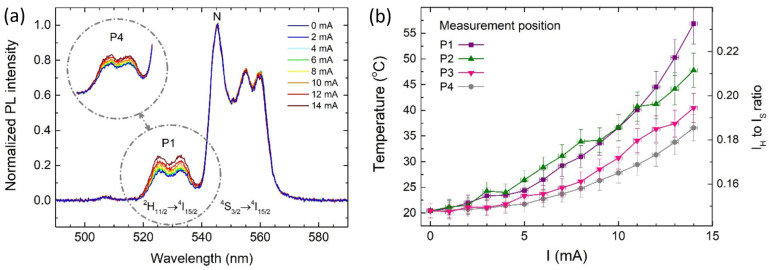
(**a**) Luminescence spectra recorded at Point 1 (Point 4 in the inset) for different current intensities flowing through the heating element. The spectra were normalized at 545 nm (N). (**b**) Temperature increase recorded at all measurement points as a function of current intensity.

Initially, the laser spot was focused on Position 1. The laser power was set to approximately 500 µW, allowing the luminescence signal to be recorded with a signal-to-noise ratio (SNR) of no less than 100. Notably, the laser polarization was linear and set perpendicular to the NW to prevent optical heating of the metal and avoid the activation of polariton excitations in the metallic nanowire particle. It is well known that silver nanowires can enhance the luminescent response of emitters, which we aimed to avoid in this case [22]. Luminescence spectra were then recorded for successive current values flowing through the microcircuit, starting from I_0_ = 0 mA (reference) and reaching up to I_max_ = 14 mA in 2 mA increments. The high sensitivity of the detection systems enabled high-quality spectra to be recorded with an acquisition time of 5 s. The same procedure was repeated for the subsequent measurement points (2–4) located in the radiator section of the microcircuit. As an example, the luminescence spectra recorded for the NC located at Position 1 are shown in Figure 5a. It is noteworthy that the increase of the current, which results in a rise of the microcircuit temperature, leads to a systematic increase of the intensity of the H-band, which is consistent with the theoretical model developed for the Er^3+^ system exposed to heat [22]. At the same time, the shape and intensity of the S-band remain unchanged.

By analyzing the relative intensities of the S- and H-bands and applying Equation (1), the temperature of the micro-heater was determined, and successive current intensities are plotted in Figure 5b. A monotonic increase in the heater temperature is observed with increasing current, reaching a maximum temperature of T_1_ = 57 °C at I = 14 mA. The measurements at subsequent positions indicate maximum values of T_2_ = 48 °C, T_3_ = 41 °C, and T_4_ = 37 °C for Positions 2–4, respectively. For comparison, the spectra obtained for Position 4 are presented in the inset of Figure 5a. The differences in the intensities of the spectra obtained for the nanocrystals at Positions 1 and 4 are visible, and the high SNR allows for a clear conclusion that the temperature decreases monotonously along the nanowire. We associated this decrease with heat dissipation through the radiator, with a temperature difference of ΔT = 20 °C over a distance of approximately 15 µm. Due to the nanoscale size of the probes, this method holds significant potential for the non-invasive imaging of heat propagation and dissipation processes in microscopic or submicroscopic electronic systems. The application of near-field detection would enable the thermal imaging of electronic circuits with spatial resolution determined by the size of the luminescence probe, which is of the order of a few nanometers [32].

## 4. Summary

The high-spatial-resolution temperature mapping of integrated electronic circuits is a significant technological challenge. In this study, we presented an optical, remote method for monitoring the temperature distribution in electrical microcircuits by utilizing luminescent nanoparticle probes, confocal microscopy, and spectroscopy techniques. We first developed a model electrical microcircuit based on a single silver nanowire to achieve this. Using standard sample preparation techniques, we defined a heating section and a cooling area within the same circuit. The heater temperature was precisely controlled using the Joule effect, which refers to the heating of a conductor when an electric current passes through it. Our results demonstrate that the temperature of such a model microcircuit can be monitored with a sensitivity of approximately 1% K^−1^ using individual nanocrystals activated with Er^3+^ and Yb^3+^ ions. Furthermore, we showed that it is possible to map the temperature distribution near a microscopic radiator and even discretely image the heat dissipation process, with spatial resolution limited only by diffraction.

## Data Availability

Data are contained within the article and Supplementary Materials.

## Supplementary Materials

The following supporting information can be downloaded at: https://www.mdpi.com/article/10.3390/nano14241985/s1, Figure S1. TEM images of NaYF_4_:Er^3+^/Yb^3+^ nanocrystals: (a) as-synthesized core and (b) core@shell nanocrystals. Figure S2. Statistical distribution of NaYF_4_:Er^3+^/Yb^3+^ nanocrystals diameter. Figure S3. X-ray diffraction patterns of NaYF_4_:Er^3+^/Yb^3+^ doped core and core@shell nanocrystals. Figure S4. Statistical distribution of Ag nanowires diameter.
