# Supplementary material for: Luminescent Nanocrystal Probes for Monitoring Temperature and Thermal Energy Dissipation of Electrical Microcircuit
Source: Nanomaterials (Basel). 2024 Dec 11;14(24):1985. doi: 10.3390/nano14241985 (PMC11728609; doi:10.3390/nano14241985)
Supplement: Supplementary file 1 [file nanomaterials-14-01985-s001.zip › nanomaterials-3330399-supplementary.pdf]

Supplementary information for:

Luminescent Nanocrystal Probes for Monitoring Temperature and Thermal Energy Dissipation of Electrical Microcircuit

Dawid Jankowski [1], Kamil Wiwatowski [1], Michał Żebrowski [1], Aleksandra Pilch-Wróbel [2], Artur Bednarkiewicz [2], Sebastian Maćkowski [1] and Dawid Piątkowski [1,*]

[1] Institute of Physics, Faculty of Physics, Astronomy and Informatics, Nicolaus Copernicus University in Toruń, ul. Grudziądzka, 5, 87-100 Toruń, Poland

[2] Institute of Low Temperature and Structure Research, Polish Academy of Sciences, ul. Okólna, 2, 50-422 Wroclaw, Poland

[*] *e-mail:* dapi@fizyka.umk.pl

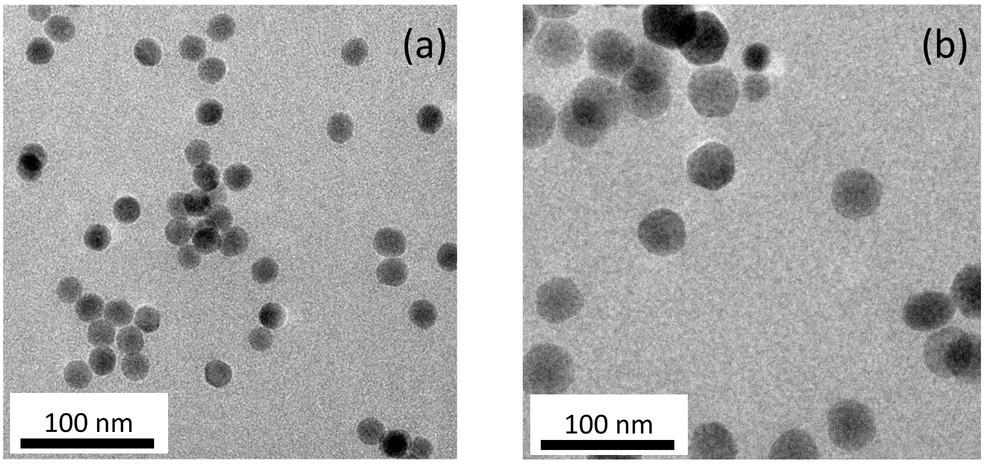

Figure S1. TEM images of $NaYF_4:Er^{3+}/Yb^{3+}$ nanocrystals: (a) as-synthesized core and (b) core@shell nanocrystals.

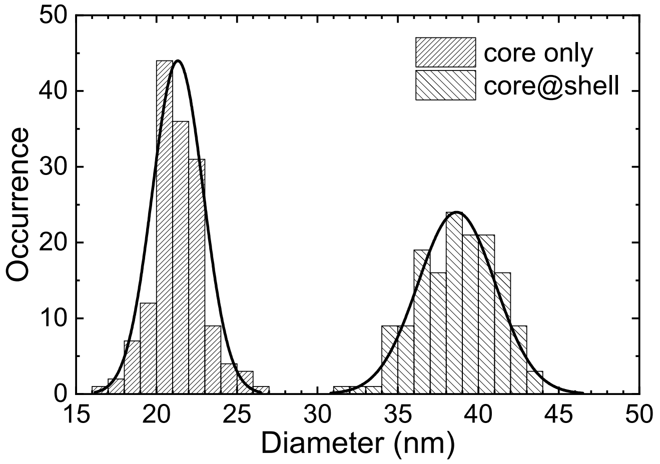

Figure S2. Statistical distribution of $NaYF_4:Er^{3+}/Yb^{3+}$ nanocrystals diameter.

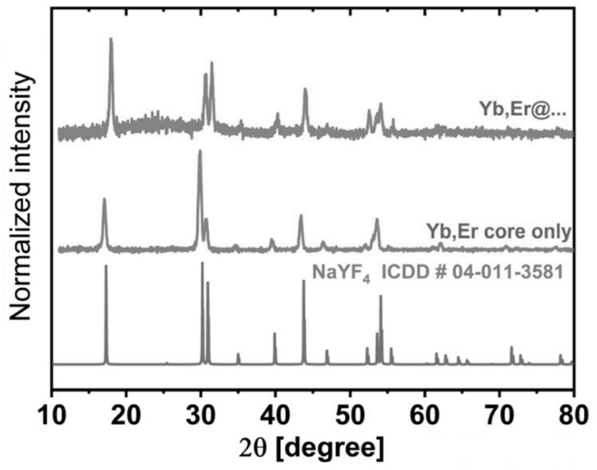

Figure S3. X-ray diffraction patterns of $NaYF_4$:Er^{3+}/Yb^{3+} doped core and core@shell nanocrystals.

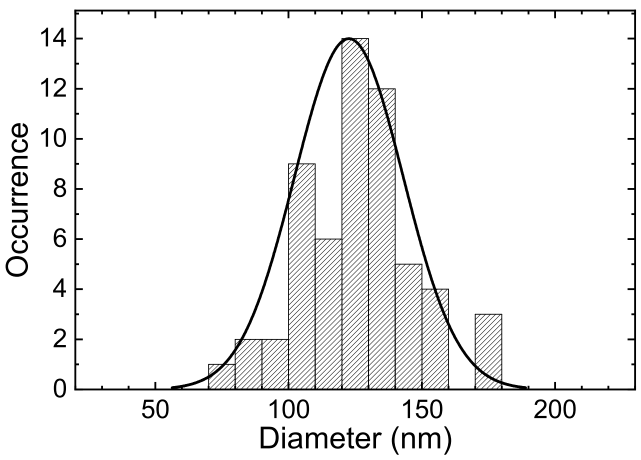

Figure S4. Statistical distribution of Ag nanowires diameter.
